# A Mapping Review of Netnography in Nursing

**DOI:** 10.1177/10497323231173794

**Published:** 2023-05-16

**Authors:** Martin Salzmann-Erikson, Henrik Eriksson

**Affiliations:** 1 Faculty of Health and Occupational Studies, Department of Caring Sciences, 3485 University of Gävle, Gävle, Sweden; 2 Department of Health Sciences, 42749 University West, Trollhättan, Sweden

**Keywords:** netnography, nursing, qualitative research, research ethics, review

## Abstract

People use the Web to seek health-related information and to discuss health issues with peers. Netnography, a qualitative research methodology, has gained the attention of researchers interested in people’s health and health issues. However, no previous reviews have accounted for how netnography is used in nursing research. The purpose of this mapping review was to generate a map of netnographic research in nursing. The search was conducted in PubMed, Academic Search Elite, the Cumulative Index to Nursing and Allied Health Literature, Medline, PsycINFO, Scopus, and Web of Science. Data were extracted from 53 original articles. The results show an increasing trend in published netnographies over time; 34% of the total sample was published in 2021. Of the total, 28% originated from Sweden, and 81% had used a covert approach. In studies in which the researchers used more participatory designs, the time spent on online forums ranged between 4 weeks and 20 months. Informed consent is found to be an issue in netnographic studies. We discuss the fact that nursing researchers have used netnography to address a wide range of research topics of concern and interest, from self-care support in an online forum for older adults to nursing students’ perspectives on effective pedagogy. In line with the digital transformation in society in general, we discuss the fact that netnography as a research methodology offers great opportunities for nurse researchers to monitor new spaces and places that presuppose online methodological knowledge.

## Introduction

In the development of qualitative research methodologies, netnography has gained the attention of researchers working in a wide range of academic fields and has been used to describe online communities (Alarcon et al., 2009; Bartl et al., 2016; Conti & Lexhagen, 2020; Henry, 2020). The principles of netnography have many similarities with the principles of its antecedent, ethnography, although the features of culture are not restricted to geospatial cultural activities; it is people’s habits and spaces that are investigated. Netnography, as described by Gambetti and Kozinets (2022), is a set of research practices aimed at comprehending the meaning behind various forms of communication and expression that occur through technology on social media platforms. As technology has a profound impact on our society, culture, and experiences, Gambetti and Kozinets conceptualize the term “technoculture” to describe the contextual elements that characterize the Web of interactions, conversations, and meaning creation on the internet. These elements include the technocultural codes and gestures that govern online behavior, the symbolic vocabularies and visual languages used to interact, the human–machine–human interfaces, the socio-technical affordances of social media platforms, and the routines and rituals that individuals establish to construct their unique online presence. Readers seeking a deeper understanding of netnography, and its core principles, should consult Kozinets’s works (Kozinets, 2002, 2015, 2019) for further information. Few literature reviews have been published on the use of netnography as a research methodology in general and none in nursing research specific. We found some subject-specific reviews, for example, from tourism (Tavakoli & Wijesinghe, 2019), and one non-subject-specific systematic review (Bartl et al., 2016). In the latter review, the authors found that netnography is increasingly being employed by researchers in various academic disciplines and that Kozinets’s methodology has had a major influence on original articles. Just as netnography has had implications for market consumer researchers and tourism, both for theory and for practice, our review intends to map the ways in which netnography has been used in nursing research to inform nurse researchers and expand their methodological repertoire.

Digital disruptions in almost every corner of society and the digital transformation have formed new spaces for cultures and micro-cultures to grow. Over the past decade, there has been huge growth in the use of social media platforms (Dixon, 2022). The significant gravitation toward willingly spending time online, in general, and on social media, in particular, is not restricted to specific countries but is instead a global phenomenon (Digital Marketing Institute, 2021). Social media come with opportunities. As spaces, the platforms help people with similar interests communicate and interact over vast geographical distances. However, mental health issues have been associated with social media use (Braghieri et al., 2022; Liu et al., 2019). Not only have social withdrawal and addictive behaviors received attention as a result of using social media applications, but the environment has also been described to include bullying, transgressions, and threats (David et al., 2018; Zhu et al., 2021). On the other hand, data from several studies have shown that social media play a pivotal supportive role among people who share everyday-life concerns and problems (Baker & Yang, 2018; Boursier et al., 2020; Selkie et al., 2020). The existing nursing research—which focuses on humans, health, environment, and nursing/caring—recognizes the critical role social media have played, as spaces, in discussing health and disease-related issues.

Our understanding of spaces and places is rooted in the work of human geographer Edward Relph (2008), who, in his book “Place and Placelessness,” posits that places are not exclusively physical locations but are also shaped by the social and cultural experiences of those who occupy them. Similarly, geographer Doreen Massey, in her book “For Space” (2005), posits that spaces are not solely physical entities but are also shaped by the social relations and power dynamics that exist within them. In netnography, the concepts of spaces and places can be applied to the study of online communities and virtual environments. For instance, a discussion forum may be considered a space; however, the memories and emotions associated with that forum by the individuals who use it, such as a place to gather with friends, would qualify it as a place. Spaces and places are determined by the interaction between the physical environment and the social and cultural experiences of those who occupy them. Thus, understanding this relationship is crucial for comprehending how people use, experience, and shape the landscapes around them. Many studies have shown how people support each other’s mental health issues, such as post-traumatic stress, anxiety, and depression, on online venues such as forum discussions and social media (Elnaggar et al., 2020; Salzmann-Erikson & Hiçdurmaz, 2017; Seabrook et al., 2016). Supportive activities on online fora were conceptualized in a framework analysis called cyber-nursing (Eriksson & Salzmann-Erikson, 2016a). That framework recognizes the fact that self-care practices take place in virtual environments, where people interact and support each other as temporary experts. However, the non-professional forms of *support and advising* that take place in virtual environments have been questioned because they are not evidence based. Moreover, how these online support strategies and health experts operate is far from fully understood. Thus, scholars have stressed the importance of monitoring these environments and supportive activities (Salzmann-Erikson & Eriksson, 2021) because such monitoring may inform nurses’ practice. Hence, netnography has become a way to increase our insights into and knowledge about nursing-relevant matters. Just as everyday living has moved toward online venues, the methodologies used to study people in their *natural settings* need to keep up with such transitions.

The impact of netnography as a methodology in nursing is understudied, particularly concerning what kinds of studies have been conducted and what research questions have been asked thanks to this method. This led us to pose the following questions: What are the characteristics of publications that use netnography in nursing and nursing-relevant research? What populations are being studied? How do authors deal with ethical considerations? What areas of nursing are in focus? Hence, the purpose of this mapping review was to generate a map of netnographic research in nursing.

## Methods

The mapping review method was selected because it is useful when resources are limited and the research question is not as detailed as in scoping, rapid, or systematic reviews (Grant & Booth, 2009; Parkhill et al., 2011). The purpose of a mapping review is to project trends on a specific topic and to describe the main characteristics of the studies included. Mapping reviews have the potential to guide clinicians, policymakers, and researchers in nursing, as highlighted by Buchholz and Dickins (2023). A key distinction between scoping and mapping reviews is that the latter provides an overview of the research landscape as opposed to synthesizing the findings of the studies. PROSPERO is a database that compiles information on systematic reviews, rapid reviews, and umbrella reviews that have been prospectively registered in the field of health and social care. However, PROSPERO does not accept registrations for scoping reviews and general literature reviews; thus, this mapping review is not eligible for inclusion in the database.

### Search Strategy

**Figure 1.**
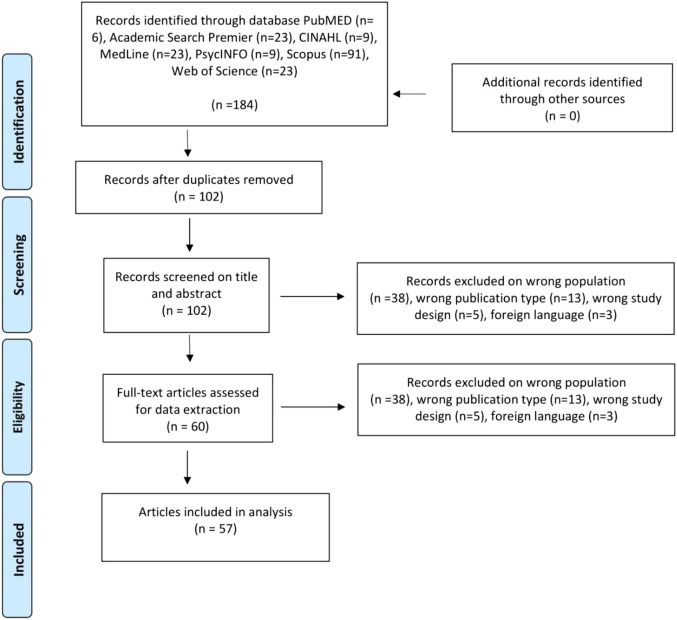
PRISMA 2009 flow diagram.

Several (*N* = 7) databases were selected based on their relevance to nursing research as well as to cover research areas of great relevance to nursing research. The selected databases were PubMed, Academic Search Elite, the Cumulative Index to Nursing and Allied Health Literature (CINAHL), Medline, PsycINFO, Scopus, and Web of Science. Two exposures were used in all databases. One exposure included the keywords *netnography* and *netnographic,* and the other exposure included the keywords *nurse*, *nurses*, *nursing*, and *nursing care*. The Boolean term OR was used within the same exposure to broaden the search, and then the two exposures were searched using the Boolean term AND to limit the search. The search was executed with both researchers present between 12 September 2022, 9:15 a.m. UTC+2, and 12 September 2022, 9:50 a.m. UTC+2. The search in all seven databases resulted in 184 records (see PRISMA flow diagram in Figure 1 for details). All 184 records were exported as *.ris and *.nbib files and then imported into a freely available review manager software.

### Selection of Sources

Based on a brief screening of the 184 articles, we came to realize that we were not in complete agreement about what should be considered nursing research. Some articles covered traditional topics in nursing research, such as service users’ experiences of receiving advice from telenurses, and these articles were agreed upon to be included. However, other articles addressed topics that could be relevant to nursing research but were not typical, such as narratives about sexual abstinence or doping use among athletes. Thus, we created an index based on our internal discussions. Working inductively, we tentatively sketched out different categories from the discussions based on a 20% (*n* = 36) sample of articles. The first category was articles that focused on topics explicitly related to nursing practice, nurses’ work life, nursing education, or nurses’ health, perceptions, etc. The second category was articles that addressed healthcare or health services, but not explicitly from the perspective of nurses, and the third category was articles addressing a medical and/or mental condition or impairment. Then we used the three categories to deductively assign the remaining articles to those categories. The latter step was performed by both authors individually. Afterward, we met for another discussion and found that we had no conflicting categorizations. The articles we assessed as belonging to level A, B, or C should be included, and those assessed as level D should be excluded. This resulted in the creation of the Nursing Research-Relevance Assessment Index (NR-RAI) (see Table 1). The NR-RAI was pilot tested on four articles, which were blinded reviewed by the two authors, and then unblinded to evaluate its consistency.

**Table 1.** Nursing Research-Relevance Assessment Index (NR-RAI).

Nursing Relevance Level A (Include)	Nursing Relevance Level B (Include)	Nursing Relevance Level C (Include)	Nursing Relevance Level D (Exclude)
Articles that were published in a nursing journal (indexed as a nursing journal or having “nursing” or “nurses” in the journal’s title).	Articles that address healthcare or health services, but not specifically from a nursing perspective.	Articles that do not relate to any specific health issue or diagnosis, illness or impairment, or deterioration of health, but that do address everyday life and the life course and its concerns.	Articles with no relevance to nursing in ways that are described in levels A–C.
Articles with a title that explicitly addressed nursing or nurses.	Articles that address disease, diagnosis, health condition, and impairment that might become relevant for nurses in their profession or in research.		
Articles where the first author was a nurse.	Articles that attract nurses based on their profession as nurses.		
The article focused on any topic related to nursing practice, work life, education, nurses’ health, etc.			
Example 1: Nursing students’ perception of effective pedagogy.	Example 1: Attitudes toward disordered eating in the rock-climbing community.	Example 1: Discussions about parental leave in relation to work lives and the private life of new fathers.	Example 1: How netnography can be adapted to research service experiences in virtual and augmented environments, which include and overlap with the notion of a Metaverse.
Example 2: Callers’ perception of national telephone advice nursing service.	Example 2: How parents of children with cancer seek information and support in online communities.	Example 2: Describing the nature of breastfeeding support that members seek and receive via closed Facebook groups.	Example 2: Identifying dimensions of consumer-perceived brand authenticity in the early stages of the development of an authentic brand.

To identify the inclusion criterion, PICO (Participants-Intervention-Comparison-Outcome) is advised in systematic reviews, but is not suitable in other kinds of reviews. Instead, we used Participants-Context-Concept (PCC) (Joanna Briggs Institute, 2014).

#### Population

We considered articles focusing on any population regardless of age, sex, marital status, socioeconomic status, or any other conceivable characteristic.

#### Concept/Phenomenon of Interest

Articles should state explicitly that the study was a “netnography” or a “netnographic study.” This information should be found in the title, abstract, and keywords—or be explicitly specified in the method section. According to Kozinets (2015), the term netnography is sometimes used offhand where other concepts would better suit the actual work, that is, “virtual ethnography” or “online research.” We did not, however, assess the methodological orthodoxy.

#### Context

##### Subject

The articles selected for this study should pertain to research that is relevant to the field of nursing. As there is no established consensus among nursing researchers regarding the boundaries of nursing research or what constitutes research that is relevant to nursing, we developed the NR-RAI to guide our data selection.

The criteria for selecting the articles were as follows: (a) original/empirical research, (b) use of netnographic methods, (c) written in English, (d) any publication years, (e) published in peer-reviewed journals, and (f) nursing research or relevant to nursing research (scoring level A to C on NR-RAI, see Table 1). Exclusion criteria were (a) review articles, (b) editorials, (c) letters to the editor, and (d) not nursing research or not relevant to nursing research (scoring level D on NR-RAI).

Quality assessment was not conducted because it is not needed in mapping reviews (Grant & Booth, 2009). After duplications were removed, the 102 articles were blind screened by both authors and assessed based on title and abstract. We agreed on the inclusion/exclusion of 79% of articles (45% agreement on includes and 36% agreement on excludes). 21% (*n* = 17) of the records were tagged as conflicting assessments, meaning that we disagreed on inclusion/exclusion. The results were unblinded, so we were able to view the conflicting records. We discussed the conflicting records one by one and reached agreement on all records, such that no conflicting records remained. All articles were downloaded into full-text and uploaded into Rayyan so that we could access the data simultaneously.

### Data Extraction

We extracted all relevant data from the articles and stored them in a screening template we created in Excel. We extracted the data from about 30 articles. The following data were extracted from the article: author(s), year, country, journal, aim/purpose, method, source of data, data collection, sample and participants, ethical vetting and/or ethical considerations, Open Access publication, and key findings. Throughout the process, we discussed the template’s issues and shortcomings and made minor adjustments. When both authors had completed the extraction of data, we merged the templates into one—the dataset (Supplementary A). We discussed the extracted data at several meetings and agreed on how to present the results in a descriptive and summative manner. Since this mapping review only encompasses previously published articles, ethical approval was not necessary.

## Results

### Characteristics of the Articles

The total dataset was based on 53 articles (100%). There is a clear upward trend in the popularity of netnographic studies, as the number of publications has increased in recent years. Between 2011 and 2016, 11 studies were published, while 36 studies were published during the past 5 years (authors’ note: the search was conducted in September 2022; thus, it is likely that even more articles will be published during the remaining nearly 5 months). The most active year was 2021, with 10 publications (19.0%). Regarding the journals in which the articles were published, this varied greatly, as 73.6% (*n* = 39) of the articles were published in different journals. Six journals had more than one publication, compared to 43 journals which only had one publication each. The most frequent journal to be included in the dataset was *Nursing Inquiry*, in which four articles (7.5%) were published, followed by *Qualitative Health Research* (*n* = 3, 5.6%).

Eight articles (15.1%) were single-author articles, and 16 (30.2%) articles were two-author collaborations. Thus, 29 articles (54.7%) had 3 or more authors. The articles originated primarily from Sweden, the UK, the USA, and Australia: Sweden (28.3%, *n* = 15), the USA (11.3%, *n* = 6), the UK (15.1%, *n* = 8), and Australia (13.2%, *n* = 7). We also extracted data on accessibility of the articles published as Open Access or as paywalls. 56.6% (*n* = 30) were published as Open Access (either in Open Access journals or in hybrid journals where the authors have paid for Open Access). Three of the accessible articles were only available in manuscript form due to the publishers’ embargo policy.

### Relevance to Nursing

Based on an assessment of the articles’ relevance to nursing according to the NR-RAI (see Table 1), 21/53 articles (39.6%) were assessed as belonging to level A, 18/53 (34.0%) as belonging to level B, and 14/53 (26.4%) as belonging to level C. As for the typology of netnographic approaches used, the analysis confirms the major impact of Kozinets’s methodology of netnography, with 49 articles (92.5%) explicitly defining their step-by-step description with reference to Kozinets’s framing of netnography as “doing ethnographic research online,” which was also the title of Kozinets’s (2010) book. The few studies that did not take Kozinets’s netnography approach as a point of departure presented their methods as discourse analysis, thematic analysis, or qualitative descriptive method. Some studies (*n* = 3) have extended Kozinets’s methodology to also include a protocol, which was originally developed to be put in practice in nursing research (Salzmann-Erikson & Eriksson, 2012). The number of articles with a cross-sectional design was 18 (34.0%), and most of the approaches included observational study designs.

Reconciled with that design is the covert presence of a researcher and a non-consent approach to data collection. When analyzing the articles on an aggregated level, we find a gradual shift in design toward interventions the longer time the researchers spend on collecting data. In contrast, other articles demonstrated how researchers were active and overtly participated in online discussions (Andréasson et al., 2018; Wallace et al., 2018), collecting participant-co-produced online data (Cano-Hila & Argemí-Baldich, 2021; Numer et al., 2022). Moreover, the explicit aim of online ethnographic fieldwork is to include an interventional design. This shift in research design also implies a shift toward an overt presence and obtained consent from participants. Articles that presented results from an online data collection with an extended overt researcher presence varied greatly concerning the time researchers spent online. Four articles (Alang & Fomotar, 2015; Cuomo et al., 2020; Giles et al., 2015; Gün & Şenol, 2019) reported 2 months of presence, and others reported 4 weeks (Bridges et al., 2018), 4 months (Cano-Hila & Argemí-Baldich, 2021), 6 months (Wilson-Nash, 2022), 10 months (Vale et al., 2019), 1 year (Baptista et al., 2021), 15 months (Wallace et al., 2018), and 20 months (Dehkhoda et al., 2020). Based on this, we can overview the methodological repertoire and conclude that it represents a continuum of approaches in many respects.

**Figure 2.**
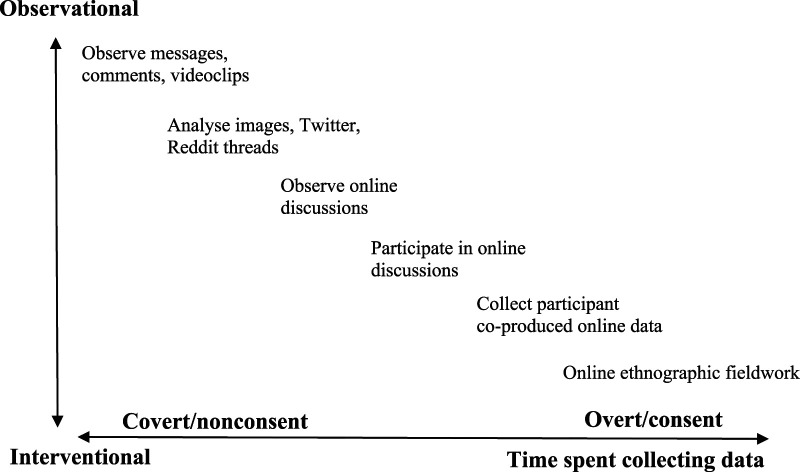
Map of characteristics in nursing netnographies.

As shown in Figure 2, there are no distinct endpoints in the characteristics of ethnographies; rather, they can be seen as existing along a continuum of observational and interventional approaches as well as a continuum of time, including researcher roles and ethical approaches.

### Reasoning About Ethical Matters

Of the total number of articles, 26.4% (*n* = 14) reported that their study had been reviewed by an ethics board (Alang & Fomotar, 2015; Andréasson et al., 2018; Aragão et al., 2018; Boursier et al., 2022; Cano-Hila & Argemí-Baldich, 2021; Ellinghaus et al., 2021; Gün & Şenol, 2019; Kendal et al., 2017; Lawless et al., 2020, 2022; Moura & Aschemann-Witzel, 2021; Schuman et al., 2019; Vale et al., 2019; Wallace et al., 2018), while 56.6% (*n* = 30) reported that their study had not been reviewed (Ari & Mari, 2021; Baptista et al., 2021; Bayen et al., 2021; Bîră et al., 2020; Björkman & Salzmann-Erikson, 2018; Botelle & Willott, 2020; Cuomo et al., 2020; Eriksson et al., 2014; Eriksson & Salzmann-Erikson, 2013, 2016b, 2018; Gatrell, 2019; Holmgren et al., 2018; Johansson & Andreasson, 2017; Keeling et al., 2015; Liang & Scammon, 2011; Manning Hutson et al., 2022; Nemec et al., 2018; Nimrod, 2011; Numer et al., 2022; Poppi, 2021; Salzmann-Erikson, 2016, 2017; Salzmann-Erikson & Eriksson, 2011; Saxena et al., 2021; Song, 2020; Strand, 2022, p. 202; Strand & Gustafsson, 2020; Thunborg & Salzmann-Erikson, 2017; Van Hout & Hearne, 2014); four articles reported that they had applied to an ethics review board (ERB) but had been deemed exempt because their work was not considered human subjects research (De Gagne et al., 2021; Giles et al., 2015; Litchman et al., 2019; Roland et al., 2017). The remaining did not report anything concerning ethical approval. In agreement with the ERBs’ decisions on exemption, some authors argued that approval from an ERB was not necessary because the data consisted of freely accessible text, no interaction occurred, and the study was cross-sectional. The authors supported this argument by providing references to articles that have debated ethics in netnographic studies. We explored to what extent the data collection in the articles was covert or overt. In some cases, but not all, this was stated explicitly; hence, we sometimes had to agree on which category—covert or overt—to assign specific articles to. We determined that 43 (81.1%) articles had used a covert approach and the remaining 10 (18.9%) an overt approach, though two of those articles included both a covert and an overt phase in the data collection.

Another methodological aspect that authors should consider is whether or not to seek permission to use the data from those who have produced the text online. Even though it is outside the aim and scope of this mapping review to statistically verify any correlations, it seemed that those who argued for not applying for ERB vetting, for the previously mentioned reasons, also argued for not seeking permission from online users. Typical reasoning is exemplified in the article by Ellinghouse et al. (2021):

The study was therefore not considered human subject research and consent was not required, a common perspective in Internet research scholarship. The current study was purely cross-sectional and observational, analyzing data from a publically available source without any intervention or interaction with forum posters. (p. 2)

Reasoning about consent did not only draw on legal jurisdictions but also on the challenges associated with obtaining consent when users do not use their legal names or are no longer active forum participants and therefore not reachable. The latter was illustrated in the article by Johansson and Andreasson (2017), who tried to reach out using posters to obtain consent but were unable to get in contact with them. However, some authors have obtained consent from forum participants (Andréasson et al., 2018; Cano-Hila & Argemí-Baldich, 2021; Fayn et al., 2021). Consent was treated slightly differently by Alang and Fomotar (2015), who used a covert approach to data collection and did not obtain permission from forum participants, but instead applied for approval from an ERB and obtained permission from the forum moderator. Although the well-acknowledged norm in netnography is not to obtain approval or consent from an ERB, ethical consideration was far from overlooked. The need to protect the online contributors’ identity was emphasized in several articles (Andréasson et al., 2018; Bîră et al., 2020; Ellinghaus et al., 2021; Johansson & Andreasson, 2017; Manning Hutson et al., 2022; Salzmann-Erikson, 2017; Salzmann-Erikson & Eriksson, 2013; Thunborg & Salzmann-Erikson, 2017). For example, fictional names were used when referring to a specific poster (Björkman & Salzmann-Erikson, 2018), and other information that could disclose the poster’s identity was modified (Botelle & Willott, 2020). Ellinghose et al. (2021) explicitly wrote that excerpts from the data were modified to protect forum posters’ anonymity. Dehkhoda et al. (2020) argued that even though excerpts are used from public venue discussions, Facebook’s policy from 2018 does not include text in general searches and therefore minimizes exploitation.

## Discussion

The present mapping review set out to generate a map of netnographic research in nursing during the past 15 years. As shown in Figure 2, the continuum of approaches utilizing the method represents many creative and innovative research initiatives. It demonstrates that nursing scholars use netnography to address a wide range of research topics of concern and interest, from self-care support on an online forum for older adults to nursing students’ perspectives on effective pedagogy. Most of the nursing research using netnography has been greatly influenced by Kozinets’s methodology. Comparison of the findings with a previous published netnography review confirms both the heterogeneity in areas in which netnography is being used, the increased numbers of publications, and the predomination of methodological references to Kozinets’s publications (Bartl et al., 2016). This finding is not surprising, as one of Kozinets’s publications has been cited by 2455 other sources according to Scite.ai and by 5754 in Google Scholar. However, few nursing research articles have discussed and/or problematized the fact that the methodology originates from social media market research. Another key finding was that most studies investigated here used observational and cross-sectional designs. Most of the studies were not reviewed by an ERB because the authors argued ethics approval was not necessary, given that the data consisted of freely accessible text and that no interaction occurred. Moreover, what is important in ethical matters is whether or not informed consent is obtained, and issues were found in relation to anonymous online posters on forums.

### Netnography Creates New Spaces and Places for Nursing Researchers

The findings indicate that there is a powerful and transformative aspect of the netnographic method to consider, beyond it simply being another tool in the researcher’s arsenal. Gambetti and Kozinets (2022) examined the relationship between netnography and the profound impact of technology on our experiences, culture, and society, and it illustrates how the method is a product of its time, with underlying epistemological differences from traditional, text-based qualitative methods in the field, such as thematic analysis, discourse analysis, and grounded theory. Traditional text-based qualitative methods, which predate technological developments, do not take into account the impact of the internet on society and the new forms of expression, communication, and pluralism it brings. Kozinets (2019, 2002, 2015) has long advocated that society is on the cusp of something fundamentally existential, evolving in our way of being and interacting that is not captured by these traditional text-based methods. Therefore, netnography offers a new perspective of forecasting previously unknown to this field.

Yet another key aspect of the research design is ethical considerations. The results show that the discussion and handling of ethical considerations are far from a consensus-oriented approach. The literature suggests that while there have been efforts to reach a consensus, the methodology of netnography and ways of designing studies are so varied that strict, unified policies are challenging to establish, in the words of Kozinets (2015): “Ethics is a moving target” (p. 130). Our findings indicate that while netnography creates new opportunities for nursing research, it also poses ethical challenges. The principle of primum non nocere (i.e., “first, do no harm”) is highly relevant in netnographic studies; however, deontological principles may not be as applicable as in traditional biomedical research. One ethical consideration in netnography, as pointed out by Kozinets (2015), is the issue of concealment. Concealment refers to the researcher’s approach in the community, such as being a lurker or obtaining permission from members, and also to the researcher’s methodological decisions regarding whether or not to protect the participants’ identities or to what degree. Guidelines for researchers have been provided by Bruckman (2002) and later modified by Kozinets (2015). These guidelines are based on the risks to participants, and when the risks are high, participants need to be protected from exploitation and researchers may need to cloak all data that can be linked to the participants; in some studies, even seemingly anonymous avatar names need to be protected. Our sample showed that about 80% of the studies used the concealed approach.

As shown in the results, there is an ongoing upward trend in the popularity of netnographic studies. One possible explanation for this trend is the overall increase in online interactions, particularly due to the COVID-19 pandemic, which has shifted many activities requiring in-person gatherings and interactions to the internet (De’ et al., 2020). The digital transformation has created new spaces and places for cultures and micro-cultures to grow—spaces and places in which nursing activities appear through manifestations of support and self-care (Wilson-Nash, 2022). The discourse of spaces and places has been emphasized in nursing literature in the past. Nurse geographers in particular have addressed the intertwining of spaces and places, as nursing geography is not restricted to geometrical boundaries; rather, the mathematical boundaries dissolved as spaces are tied to social dimensions and identity qualities (Andrews & Shaw, 2008). Halford and Leonard (2003) put it as follows: “not only do people make spaces but spaces may be used to make people” (p. 202). The underlying epistemological assumptions of the connections between spaces and places, as emphasized by Halford and Leonard, can be succinctly summarized through a quote from Friedrich Nietzsche: “if you gaze long enough into the abyss, the abyss gazes back into you” (Nietzsche & Kaufmann, 1989, p. 88). The spaces represent living subjective matter that also unconditionally changes its people, including the researchers. Just as nurse geography is highly applicable in gerontological and geriatric nursing, in the process of moving to a nursing home (Fänge & Ivanoff, 2009), nurse geography also becomes relevant to cyber-nursing. However, due to the use of technology, the possibilities for *culture-hopping*—the ability to move across vastly separated online cultures with the click of a mouse—need to be accounted for and further investigated by cyber-nurse researchers.

**Figure 3.**
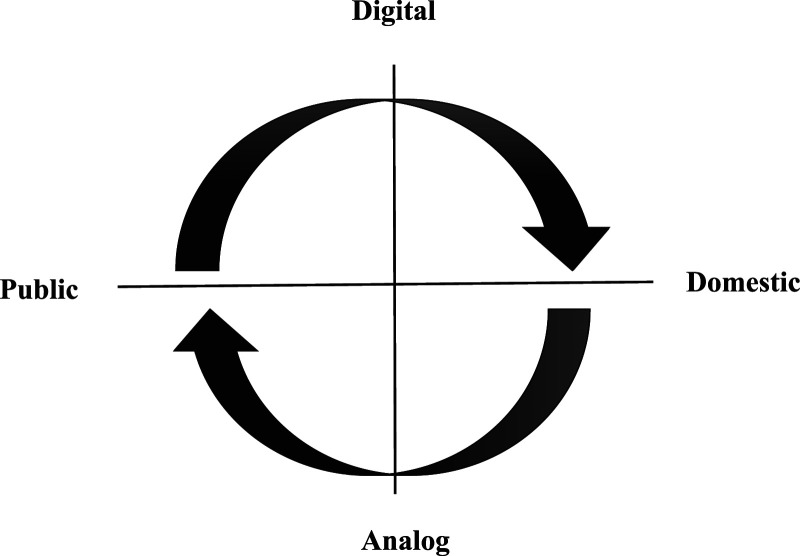
The circularity of netnographic research in available spaces.

We should also consider that, due to the ongoing rapid technological development with explosive use of social media and with internet access to over 4.9 billion users worldwide in 2020, this shift in interest in “what happens online with regards to nursing” will probably not decline; instead, we predict an exponential increase over the years to come. The vast growth of patient interactions on health forums online with a hyper-increased clinical online presence worldwide has become an important activity to monitor. As Lupton (2013) pointed out, when the clinic goes virtual, it not only moves the clinic into the home but also disperses it to every possible spatial and temporal location. The ongoing research interest in virtual environments follows a given pattern of power shifts in spaces, moving from public analog spaces, typically represented by the clinic, to digital domestic spaces, typically represented by the home. To visualize this relation between spaces, new and old, we draw attention to this four-field table of spaces:

Many of the studies included in this review specifically focus on the dialectical connection between places, often as the motivation and justification for the study. This dialectical assumption provides an ontological foundation that precedes differences in design and should be further examined. As shown in Figure 3, the presentations of content and connections in the included articles support the notion that there is a dialectical relationship between the available spaces. The clockwise arrows in the figure illustrate that when researchers are monitoring online self-care trends, the ideas—intentionally or unintentionally—spin from the domestic space to the working public places, such as the clinic, and also vice-versa when monitoring clinical experience. When monitoring these ongoing changes in spaces, the research is simultaneously influencing the transition between the spaces, underpinning the ongoing circularity of the spaces visualized in Figure 3. As pointed out by Halford and Leonard (2003), observing also means influencing this ongoing circularity of the transition of spaces. The results highlight the strong link between the increasing use of online clinical services and the proliferation of virtual self-care platforms for health guidance. As Lupton (2013) observed, these platforms are accessible across a wide range of spatial and temporal locations. We can conclude that netnography itself is part of this interconnected relationship, which—through its scientific approach—disperses content across the spaces it claims to study.

### Considerations Arising for the Next Generation of Nursing Netnographers

At the beginning of netnography (in the 1990s), the Web was still in its infancy. Users were only able to read and consume information: Web 1.0. However, with Web 2.0, users were able to communicate in two directions, meaning that users were not restricted to reading but could also write on the Web, communicating synchronously or asynchronously (Hilty et al., 2021; Salzmann-Erikson & Eriksson, 2011). Some examples of early Web 2.0 applications were GeoCities and AOL. Some years later, social media platforms such as MySpace, Facebook, YouTube, Flickr, and Instagram experienced mass adoption. These spaces were investigated by market researchers (Patino et al., 2012). Web 2.0 paved the way for nurse netnographers to use the method flexibly, as demonstrated in our results. Even so, netnographies have been restricted to investigate Web 2.0. As users of Web 2.0 were able to both read and write, users of Web 3.0 are able to read, write, and *own,* via a decentralized ledger that, due to advanced cryptography, cannot be tampered with. As a consequence of enabling ownership via the blockchain technology—being the infrastructure of Web 3.0—and decentralization, organizations are being built on blockchain technology, and decentralized autonomous organizations (DAOs) are being built (El Faqir, Arroyo & Hassan, 2020). In DAOs, the power balance shifts and ends up in the hands of the community and owner, rather than traditional organizations with a board. From the employment of smart contracts to a cryptographic way to cut the need for a third party or a middle-man, DAOs can provide community members with trust, governance, and decision-making. Based on the rising interest in DAOs as a cultural space in Web 3.0, it is likely that organizations of relevance to nursing will make use of its pros, for example, patient associations.

Just as Kozinets (2015) argued that ethics is a moving target, we also recognize that the future of netnography is dependent on the ever-evolving technological landscape. As technology continues to develop and create new virtual spaces, individuals (or avatars) interact and form new memories and emotions within these environments. Therefore, we argue that the future of netnography is tied to the progression of the Web and the emergence of Web 3.0. Given that netnography is philosophically firmly rooted in an anthropologic ontology and constructivist epistemology, we predict the need for using its inherent characteristics, such as flexibility and robustness, in contrast to atheoretical methods such as thematic analysis, which are characterized by “theoretical freedom” (Braun & Clarke, 2006, p. 78). However, Thorne (1991) has cautioned nurse researchers to use methodologies derived from social science and anthropology, such as ethnography and phenomenology, due to the challenges that nurses face in achieving methodological orthodoxy with these methods. Thorne argued that “while the culture concept represents part of what we want to understand, it is never the whole. Our enterprise is therefore somewhat more ‘fuzzy’ than true ethnography” (1991, p. 190). Nevertheless, as new online venues continue to emerge, we believe that netnography is the most validated method for studying these new spaces and places and is therefore strongly recommended for use in nursing science.

### Methodological Considerations

The database searches for nursing research that has used netnography were conducted in seven databases, and year of publication was not restricted to a specific range of years. This is considered a methodological strength, especially because this is a mapping review, which has lower demands for systematic searches than does a systematic review. There is no consensus regarding what should be considered nursing research or nursing-relevant research. This was a methodological challenge that we managed by formulating the NR-RAI. This index was not validated by other nursing researchers, which would have additionally strengthened its internal validity. However, as two senior researchers in nursing research, we consider the index’s internal consistency to be good enough for the present purpose—we do not claim anything regarding the index’s external validity. Another potential limitation is that more keywords could have been used to identify more netnographic inquiries. For example, articles defining their method as “virtual ethnography” and using other similar terms were excluded from the search. The risk of missing potential studies when conducting a search is also acknowledged as the most common limitation of literature reviews (Pham et al., 2014).

## Conclusion

Netnographic research in nursing has increased in popularity over the past 15 years, with nursing scholars using the method to address a variety of research topics. Most of the nursing research using netnography has been influenced by Kozinets’s methodology and has used observational and cross-sectional designs. Many of the studies were not reviewed by an ethical review board due to the use of freely accessible text and the lack of interaction with participants, but ethical considerations, such as informed consent and protecting the anonymity of online forum posters, were found to be important. Netnography creates new spaces for nursing research but also presents ethical challenges, particularly in relation to concealment and the protection of participant data. There is a need for more standardized approaches to address these ethical and methodological issues. The increasing trend of netnographic studies may be due to the increasing use of online interaction and the digital transformation. However, more research is needed to examine the potential risks and benefits of using netnography in nursing research. Therefore, it is crucial that nursing researchers have access to a framework for analyzing health interactions over the internet that is easily accessible. The insights gained from studying health interactions on the internet provide important trajectories of knowledge in fields that may otherwise be overlooked or silenced. Since the mid-1990s, netnography has evolved and has been effectively utilized as such a framework across various disciplines. As the most validated method for studying society through the internet, it is strongly recommended for use in nursing science.

## Supplemental Material

Supplemental Material - A Mapping Review of Netnography in Nursing Click here for additional data file. Supplemental Material for A Mapping Review of Netnography in Nursing by Martin Salzmann-Erikson and Henrik Eriksson in Qualitative Health Research
